# Supplementary figures and images for: A comparative study on polyp classification using convolutional neural networks (part 2 of 2)
Source: PLoS One. 2020 Jul 30;15(7):e0236452. doi: 10.1371/journal.pone.0236452 (PMC7392235; doi:10.1371/journal.pone.0236452)

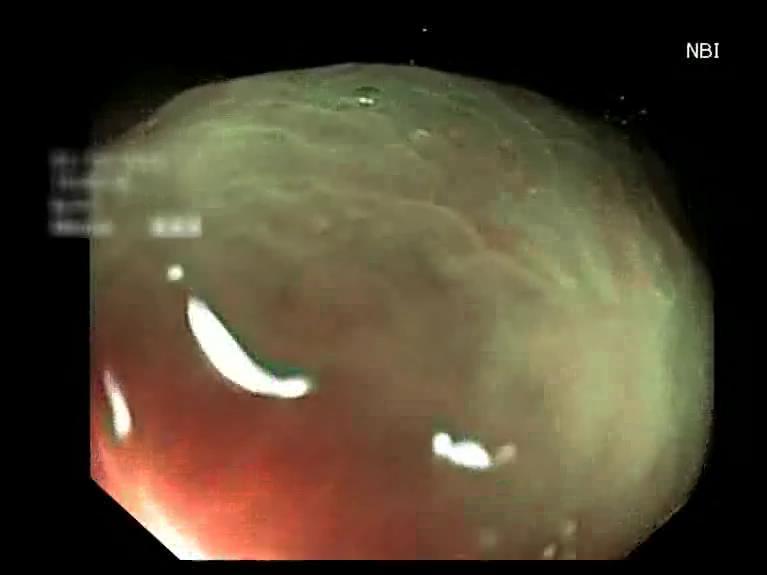

Supplement: S1 Dataset — (ZIP) [file pone.0236452.s001.zip › Dataset/set-2/Adenoma/test23_29.jpg]

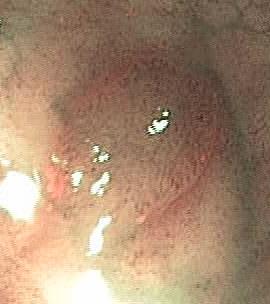

Supplement: S1 Dataset — (ZIP) [file pone.0236452.s001.zip › Dataset/set-2/Adenoma/test8_387.jpg]

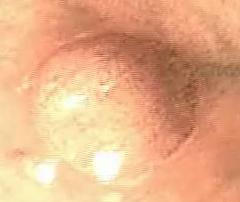

Supplement: S1 Dataset — (ZIP) [file pone.0236452.s001.zip › Dataset/set-2/Adenoma/test16_92.jpg]

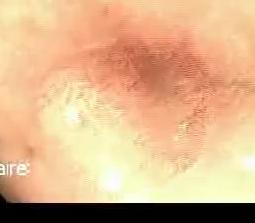

Supplement: S1 Dataset — (ZIP) [file pone.0236452.s001.zip › Dataset/set-2/Adenoma/test16_86.jpg]

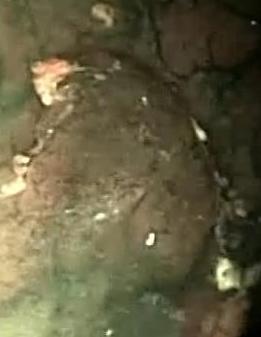

Supplement: S1 Dataset — (ZIP) [file pone.0236452.s001.zip › Dataset/set-2/Adenoma/test24_34.jpg]

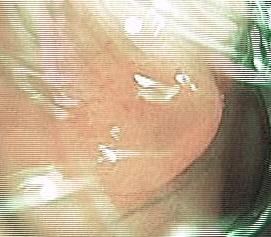

Supplement: S1 Dataset — (ZIP) [file pone.0236452.s001.zip › Dataset/set-2/Adenoma/test22_156.jpg]

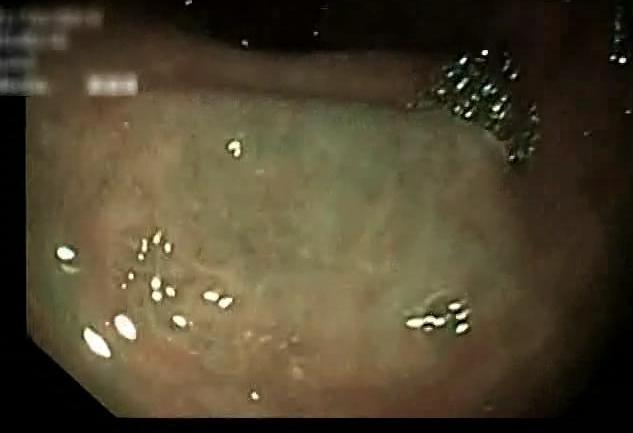

Supplement: S1 Dataset — (ZIP) [file pone.0236452.s001.zip › Dataset/set-2/Adenoma/test23_2.jpg]

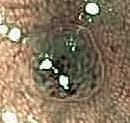

Supplement: S1 Dataset — (ZIP) [file pone.0236452.s001.zip › Dataset/set-2/Adenoma/test19_163.jpg]

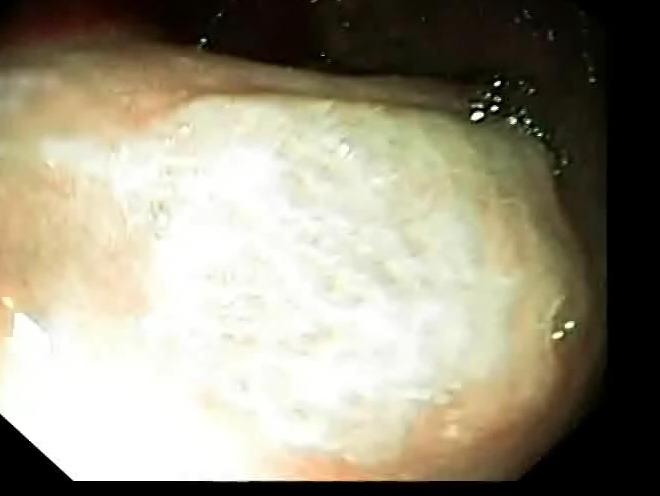

Supplement: S1 Dataset — (ZIP) [file pone.0236452.s001.zip › Dataset/set-2/Adenoma/test23_15.jpg]

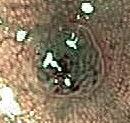

Supplement: S1 Dataset — (ZIP) [file pone.0236452.s001.zip › Dataset/set-2/Adenoma/test19_177.jpg]

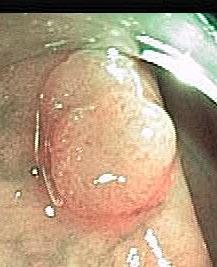

Supplement: S1 Dataset — (ZIP) [file pone.0236452.s001.zip › Dataset/set-2/Adenoma/test22_181.jpg]

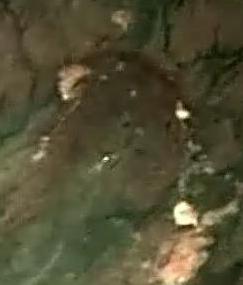

Supplement: S1 Dataset — (ZIP) [file pone.0236452.s001.zip › Dataset/set-2/Adenoma/test24_113.jpg]

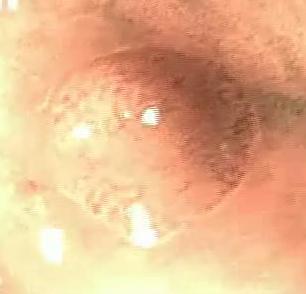

Supplement: S1 Dataset — (ZIP) [file pone.0236452.s001.zip › Dataset/set-2/Adenoma/test16_51.jpg]

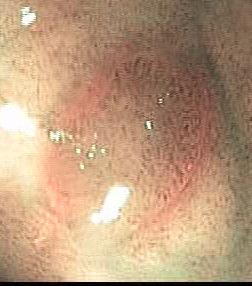

Supplement: S1 Dataset — (ZIP) [file pone.0236452.s001.zip › Dataset/set-2/Adenoma/test8_344.jpg]

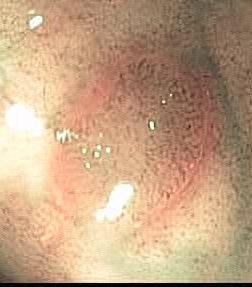

Supplement: S1 Dataset — (ZIP) [file pone.0236452.s001.zip › Dataset/set-2/Adenoma/test8_350.jpg]

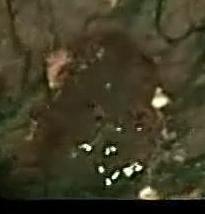

Supplement: S1 Dataset — (ZIP) [file pone.0236452.s001.zip › Dataset/set-2/Adenoma/test24_107.jpg]

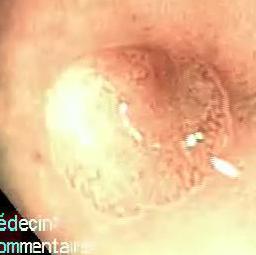

Supplement: S1 Dataset — (ZIP) [file pone.0236452.s001.zip › Dataset/set-2/Adenoma/test16_45.jpg]

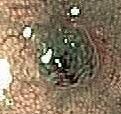

Supplement: S1 Dataset — (ZIP) [file pone.0236452.s001.zip › Dataset/set-2/Adenoma/test19_188.jpg]

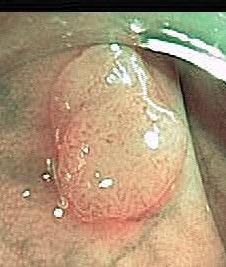

Supplement: S1 Dataset — (ZIP) [file pone.0236452.s001.zip › Dataset/set-2/Adenoma/test22_195.jpg]

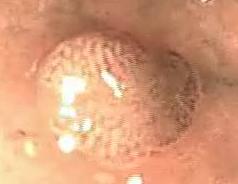

Supplement: S1 Dataset — (ZIP) [file pone.0236452.s001.zip › Dataset/set-2/Adenoma/test16_79.jpg]

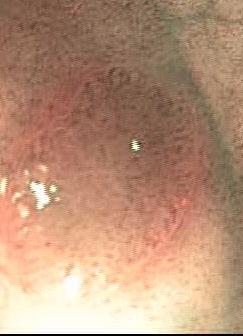

Supplement: S1 Dataset — (ZIP) [file pone.0236452.s001.zip › Dataset/set-2/Adenoma/test8_185.jpg]

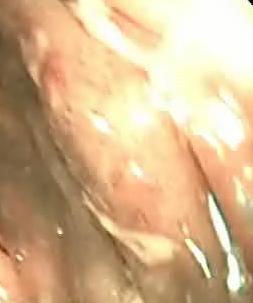

Supplement: S1 Dataset — (ZIP) [file pone.0236452.s001.zip › Dataset/set-2/Adenoma/test15_179.jpg]

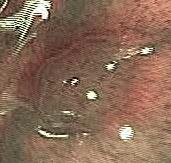

Supplement: S1 Dataset — (ZIP) [file pone.0236452.s001.zip › Dataset/set-2/Adenoma/test21_283.jpg]

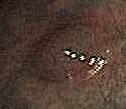

Supplement: S1 Dataset — (ZIP) [file pone.0236452.s001.zip › Dataset/set-2/Adenoma/test21_297.jpg]

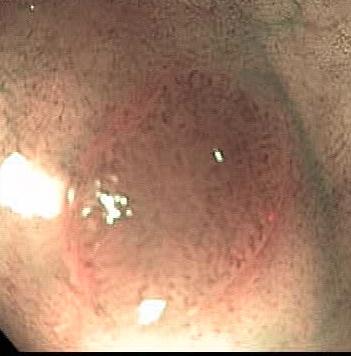

Supplement: S1 Dataset — (ZIP) [file pone.0236452.s001.zip › Dataset/set-2/Adenoma/test8_191.jpg]

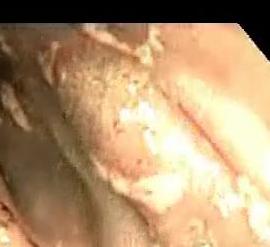

Supplement: S1 Dataset — (ZIP) [file pone.0236452.s001.zip › Dataset/set-2/Adenoma/test15_145.jpg]

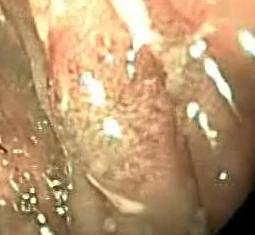

Supplement: S1 Dataset — (ZIP) [file pone.0236452.s001.zip › Dataset/set-2/Adenoma/test15_151.jpg]

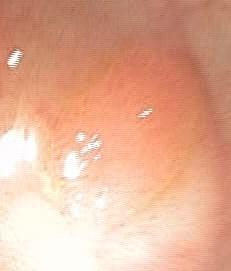

Supplement: S1 Dataset — (ZIP) [file pone.0236452.s001.zip › Dataset/set-2/Adenoma/test8_146.jpg]

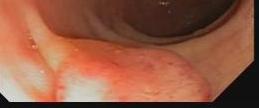

Supplement: S1 Dataset — (ZIP) [file pone.0236452.s001.zip › Dataset/set-2/Adenoma/test1_233.jpg]

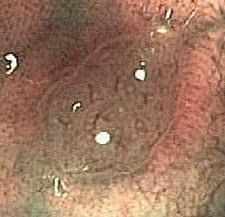

Supplement: S1 Dataset — (ZIP) [file pone.0236452.s001.zip › Dataset/set-2/Adenoma/test21_240.jpg]

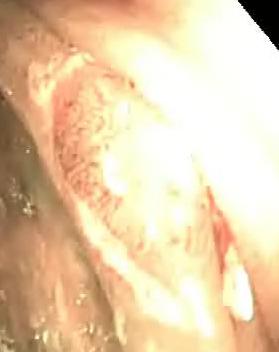

Supplement: S1 Dataset — (ZIP) [file pone.0236452.s001.zip › Dataset/set-2/Adenoma/test15_28.jpg]

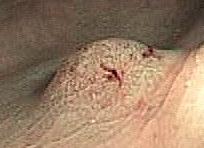

Supplement: S1 Dataset — (ZIP) [file pone.0236452.s001.zip › Dataset/set-2/Adenoma/test5_165.jpg]

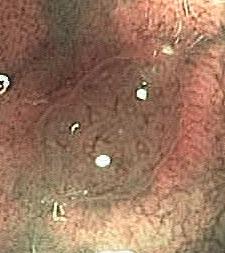

Supplement: S1 Dataset — (ZIP) [file pone.0236452.s001.zip › Dataset/set-2/Adenoma/test21_254.jpg]

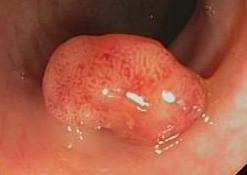

Supplement: S1 Dataset — (ZIP) [file pone.0236452.s001.zip › Dataset/set-2/Adenoma/test1_541.jpg]

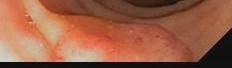

Supplement: S1 Dataset — (ZIP) [file pone.0236452.s001.zip › Dataset/set-2/Adenoma/test1_227.jpg]

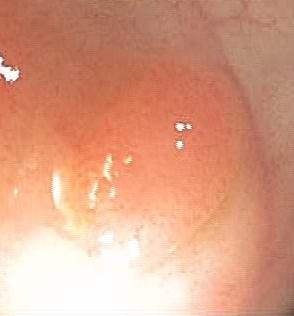

Supplement: S1 Dataset — (ZIP) [file pone.0236452.s001.zip › Dataset/set-2/Adenoma/test8_152.jpg]

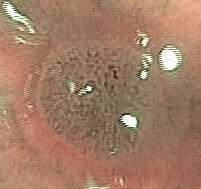

Supplement: S1 Dataset — (ZIP) [file pone.0236452.s001.zip › Dataset/set-2/Adenoma/test17_256.jpg]

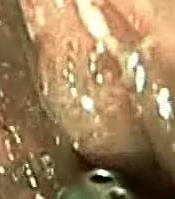

Supplement: S1 Dataset — (ZIP) [file pone.0236452.s001.zip › Dataset/set-2/Adenoma/test15_186.jpg]

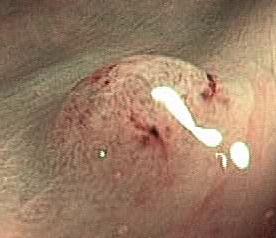

Supplement: S1 Dataset — (ZIP) [file pone.0236452.s001.zip › Dataset/set-2/Adenoma/test5_159.jpg]

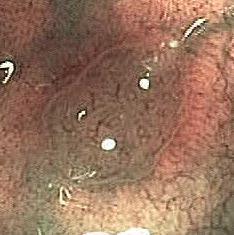

Supplement: S1 Dataset — (ZIP) [file pone.0236452.s001.zip › Dataset/set-2/Adenoma/test21_268.jpg]

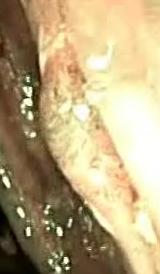

Supplement: S1 Dataset — (ZIP) [file pone.0236452.s001.zip › Dataset/set-2/Adenoma/test15_14.jpg]

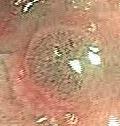

Supplement: S1 Dataset — (ZIP) [file pone.0236452.s001.zip › Dataset/set-2/Adenoma/test17_242.jpg]

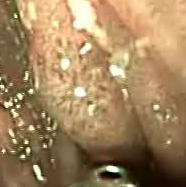

Supplement: S1 Dataset — (ZIP) [file pone.0236452.s001.zip › Dataset/set-2/Adenoma/test15_192.jpg]

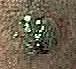

Supplement: S1 Dataset — (ZIP) [file pone.0236452.s001.zip › Dataset/set-2/Adenoma/test19_229.jpg]

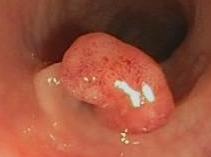

Supplement: S1 Dataset — (ZIP) [file pone.0236452.s001.zip › Dataset/set-2/Adenoma/test1_384.jpg]

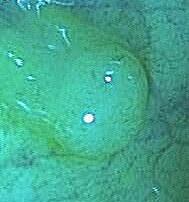

Supplement: S1 Dataset — (ZIP) [file pone.0236452.s001.zip › Dataset/set-2/Adenoma/test22_41.jpg]

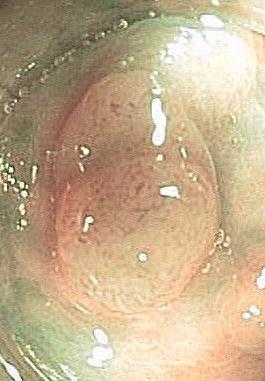

Supplement: S1 Dataset — (ZIP) [file pone.0236452.s001.zip › Dataset/set-2/Adenoma/test22_234.jpg]

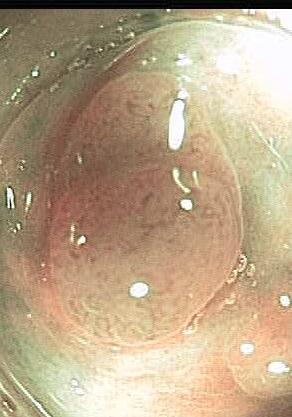

Supplement: S1 Dataset — (ZIP) [file pone.0236452.s001.zip › Dataset/set-2/Adenoma/test22_220.jpg]

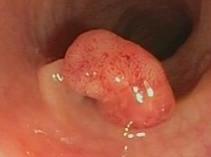

Supplement: S1 Dataset — (ZIP) [file pone.0236452.s001.zip › Dataset/set-2/Adenoma/test1_390.jpg]

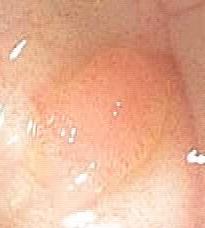

Supplement: S1 Dataset — (ZIP) [file pone.0236452.s001.zip › Dataset/set-2/Adenoma/test8_77.jpg]

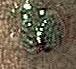

Supplement: S1 Dataset — (ZIP) [file pone.0236452.s001.zip › Dataset/set-2/Adenoma/test19_215.jpg]

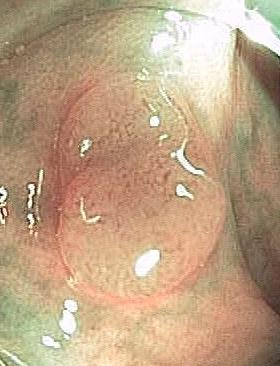

Supplement: S1 Dataset — (ZIP) [file pone.0236452.s001.zip › Dataset/set-2/Adenoma/test22_208.jpg]

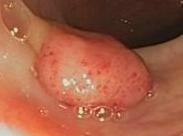

Supplement: S1 Dataset — (ZIP) [file pone.0236452.s001.zip › Dataset/set-2/Adenoma/test1_53.jpg]

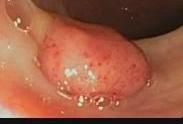

Supplement: S1 Dataset — (ZIP) [file pone.0236452.s001.zip › Dataset/set-2/Adenoma/test1_47.jpg]

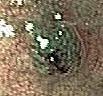

Supplement: S1 Dataset — (ZIP) [file pone.0236452.s001.zip › Dataset/set-2/Adenoma/test19_201.jpg]

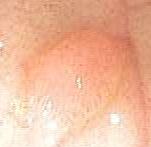

Supplement: S1 Dataset — (ZIP) [file pone.0236452.s001.zip › Dataset/set-2/Adenoma/test8_63.jpg]

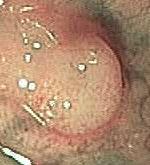

Supplement: S1 Dataset — (ZIP) [file pone.0236452.s001.zip › Dataset/set-2/Adenoma/test22_69.jpg]

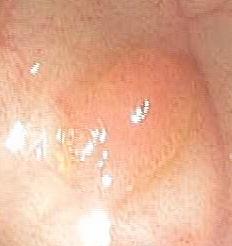

Supplement: S1 Dataset — (ZIP) [file pone.0236452.s001.zip › Dataset/set-2/Adenoma/test8_88.jpg]

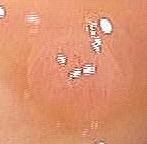

Supplement: S1 Dataset — (ZIP) [file pone.0236452.s001.zip › Dataset/set-2/Adenoma/test17_39.jpg]

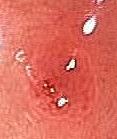

Supplement: S1 Dataset — (ZIP) [file pone.0236452.s001.zip › Dataset/set-2/Adenoma/test19_14.jpg]

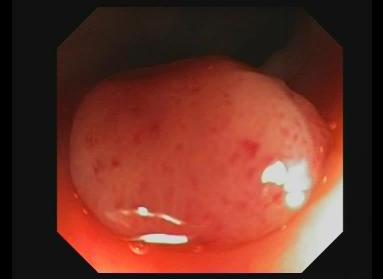

Supplement: S1 Dataset — (ZIP) [file pone.0236452.s001.zip › Dataset/set-2/Adenoma/test1_347.jpg]

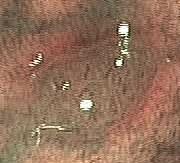

Supplement: S1 Dataset — (ZIP) [file pone.0236452.s001.zip › Dataset/set-2/Adenoma/test21_334.jpg]

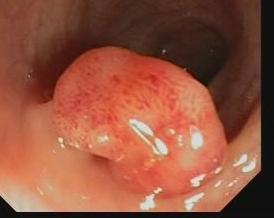

Supplement: S1 Dataset — (ZIP) [file pone.0236452.s001.zip › Dataset/set-2/Adenoma/test1_421.jpg]

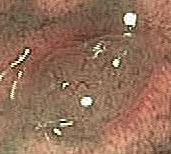

Supplement: S1 Dataset — (ZIP) [file pone.0236452.s001.zip › Dataset/set-2/Adenoma/test21_320.jpg]

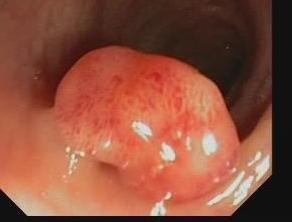

Supplement: S1 Dataset — (ZIP) [file pone.0236452.s001.zip › Dataset/set-2/Adenoma/test1_435.jpg]

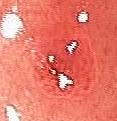

Supplement: S1 Dataset — (ZIP) [file pone.0236452.s001.zip › Dataset/set-2/Adenoma/test19_28.jpg]

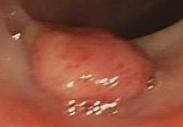

Supplement: S1 Dataset — (ZIP) [file pone.0236452.s001.zip › Dataset/set-2/Adenoma/test1_90.jpg]

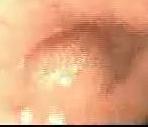

Supplement: S1 Dataset — (ZIP) [file pone.0236452.s001.zip › Dataset/set-2/Adenoma/test16_131.jpg]

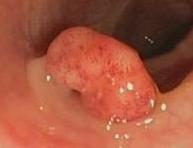

Supplement: S1 Dataset — (ZIP) [file pone.0236452.s001.zip › Dataset/set-2/Adenoma/test1_409.jpg]

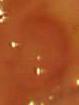

Supplement: S1 Dataset — (ZIP) [file pone.0236452.s001.zip › Dataset/set-2/Adenoma/test10_18.jpg]

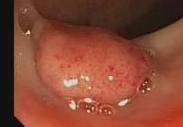

Supplement: S1 Dataset — (ZIP) [file pone.0236452.s001.zip › Dataset/set-2/Adenoma/test1_84.jpg]

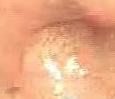

Supplement: S1 Dataset — (ZIP) [file pone.0236452.s001.zip › Dataset/set-2/Adenoma/test16_125.jpg]

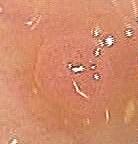

Supplement: S1 Dataset — (ZIP) [file pone.0236452.s001.zip › Dataset/set-2/Adenoma/test17_11.jpg]

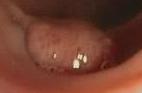

Supplement: S1 Dataset — (ZIP) [file pone.0236452.s001.zip › Dataset/set-2/Adenoma/test1_186.jpg]

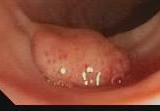

Supplement: S1 Dataset — (ZIP) [file pone.0236452.s001.zip › Dataset/set-2/Adenoma/test1_192.jpg]

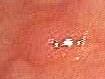

Supplement: S1 Dataset — (ZIP) [file pone.0236452.s001.zip › Dataset/set-2/Adenoma/test21_38.jpg]

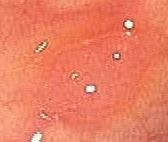

Supplement: S1 Dataset — (ZIP) [file pone.0236452.s001.zip › Dataset/set-2/Adenoma/test21_10.jpg]

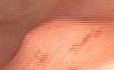

Supplement: S1 Dataset — (ZIP) [file pone.0236452.s001.zip › Dataset/set-2/Adenoma/test5_37.jpg]

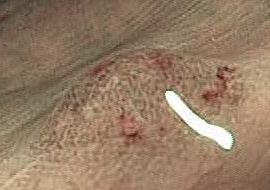

Supplement: S1 Dataset — (ZIP) [file pone.0236452.s001.zip › Dataset/set-2/Adenoma/test5_207.jpg]

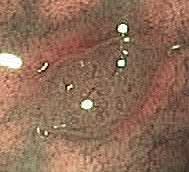

Supplement: S1 Dataset — (ZIP) [file pone.0236452.s001.zip › Dataset/set-2/Adenoma/test21_136.jpg]

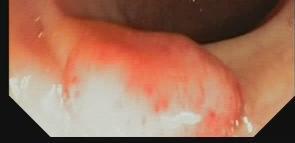

Supplement: S1 Dataset — (ZIP) [file pone.0236452.s001.zip › Dataset/set-2/Adenoma/test1_145.jpg]

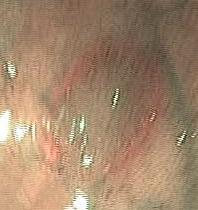

Supplement: S1 Dataset — (ZIP) [file pone.0236452.s001.zip › Dataset/set-2/Adenoma/test8_230.jpg]

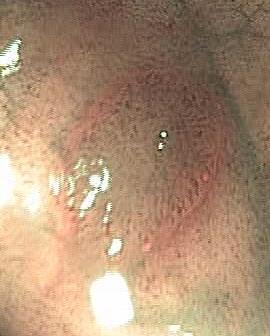

Supplement: S1 Dataset — (ZIP) [file pone.0236452.s001.zip › Dataset/set-2/Adenoma/test8_224.jpg]

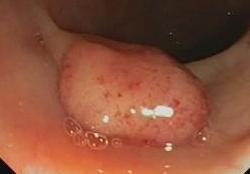

Supplement: S1 Dataset — (ZIP) [file pone.0236452.s001.zip › Dataset/set-2/Adenoma/test1_151.jpg]

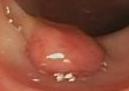

Supplement: S1 Dataset — (ZIP) [file pone.0236452.s001.zip › Dataset/set-2/Adenoma/test1_3.jpg]

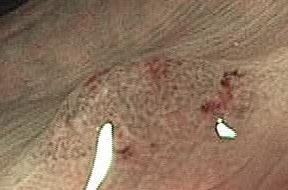

Supplement: S1 Dataset — (ZIP) [file pone.0236452.s001.zip › Dataset/set-2/Adenoma/test5_213.jpg]

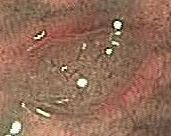

Supplement: S1 Dataset — (ZIP) [file pone.0236452.s001.zip › Dataset/set-2/Adenoma/test21_122.jpg]

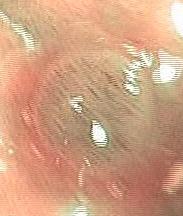

Supplement: S1 Dataset — (ZIP) [file pone.0236452.s001.zip › Dataset/set-2/Adenoma/test17_108.jpg]

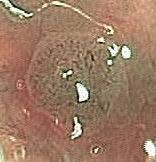

Supplement: S1 Dataset — (ZIP) [file pone.0236452.s001.zip › Dataset/set-2/Adenoma/test17_120.jpg]

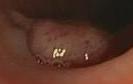

Supplement: S1 Dataset — (ZIP) [file pone.0236452.s001.zip › Dataset/set-2/Adenoma/test1_179.jpg]

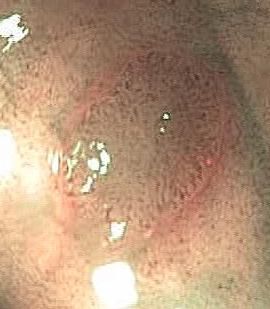

Supplement: S1 Dataset — (ZIP) [file pone.0236452.s001.zip › Dataset/set-2/Adenoma/test8_218.jpg]

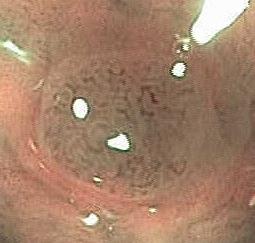

Supplement: S1 Dataset — (ZIP) [file pone.0236452.s001.zip › Dataset/set-2/Adenoma/test17_134.jpg]

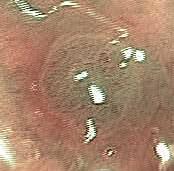

Supplement: S1 Dataset — (ZIP) [file pone.0236452.s001.zip › Dataset/set-2/Adenoma/test17_122.jpg]

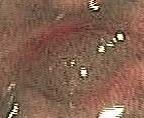

Supplement: S1 Dataset — (ZIP) [file pone.0236452.s001.zip › Dataset/set-2/Adenoma/test21_108.jpg]

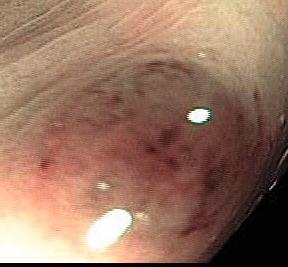

Supplement: S1 Dataset — (ZIP) [file pone.0236452.s001.zip › Dataset/set-2/Adenoma/test5_239.jpg]

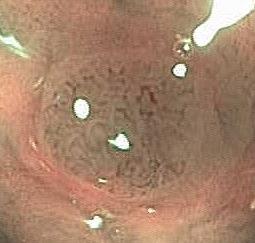

Supplement: S1 Dataset — (ZIP) [file pone.0236452.s001.zip › Dataset/set-2/Adenoma/test17_136.jpg]

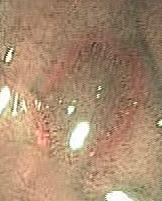

Supplement: S1 Dataset — (ZIP) [file pone.0236452.s001.zip › Dataset/set-2/Adenoma/test8_232.jpg]

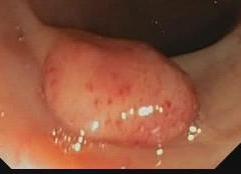

Supplement: S1 Dataset — (ZIP) [file pone.0236452.s001.zip › Dataset/set-2/Adenoma/test1_147.jpg]

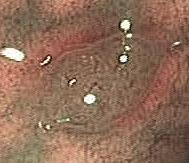

Supplement: S1 Dataset — (ZIP) [file pone.0236452.s001.zip › Dataset/set-2/Adenoma/test21_134.jpg]

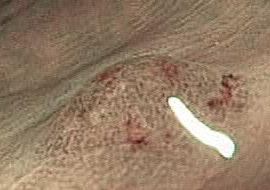

Supplement: S1 Dataset — (ZIP) [file pone.0236452.s001.zip › Dataset/set-2/Adenoma/test5_205.jpg]
