# Supplementary figures and images for: A comparative study on polyp classification using convolutional neural networks (part 1 of 2)
Source: PLoS One. 2020 Jul 30;15(7):e0236452. doi: 10.1371/journal.pone.0236452 (PMC7392235; doi:10.1371/journal.pone.0236452)

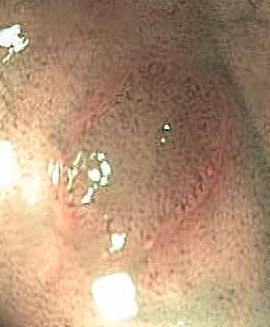

Supplement: S1 Dataset — (ZIP) [file pone.0236452.s001.zip › Dataset/set-2/Adenoma/test8_219.jpg]

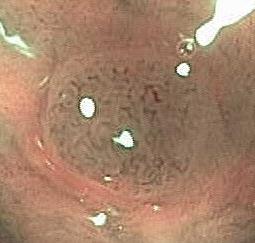

Supplement: S1 Dataset — (ZIP) [file pone.0236452.s001.zip › Dataset/set-2/Adenoma/test17_135.jpg]

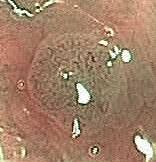

Supplement: S1 Dataset — (ZIP) [file pone.0236452.s001.zip › Dataset/set-2/Adenoma/test17_121.jpg]

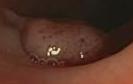

Supplement: S1 Dataset — (ZIP) [file pone.0236452.s001.zip › Dataset/set-2/Adenoma/test1_178.jpg]

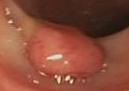

Supplement: S1 Dataset — (ZIP) [file pone.0236452.s001.zip › Dataset/set-2/Adenoma/test1_2.jpg]

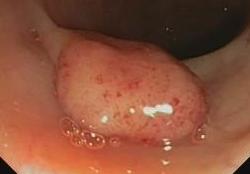

Supplement: S1 Dataset — (ZIP) [file pone.0236452.s001.zip › Dataset/set-2/Adenoma/test1_150.jpg]

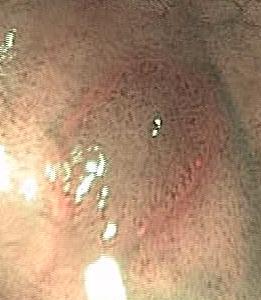

Supplement: S1 Dataset — (ZIP) [file pone.0236452.s001.zip › Dataset/set-2/Adenoma/test8_225.jpg]

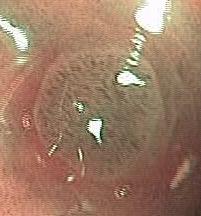

Supplement: S1 Dataset — (ZIP) [file pone.0236452.s001.zip › Dataset/set-2/Adenoma/test17_109.jpg]

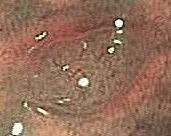

Supplement: S1 Dataset — (ZIP) [file pone.0236452.s001.zip › Dataset/set-2/Adenoma/test21_123.jpg]

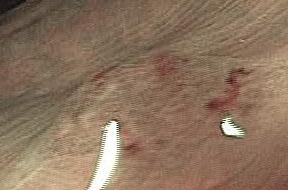

Supplement: S1 Dataset — (ZIP) [file pone.0236452.s001.zip › Dataset/set-2/Adenoma/test5_212.jpg]

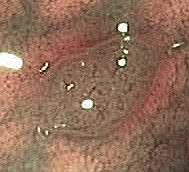

Supplement: S1 Dataset — (ZIP) [file pone.0236452.s001.zip › Dataset/set-2/Adenoma/test21_137.jpg]

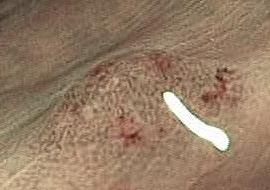

Supplement: S1 Dataset — (ZIP) [file pone.0236452.s001.zip › Dataset/set-2/Adenoma/test5_206.jpg]

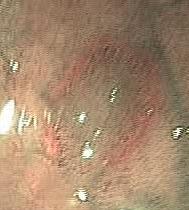

Supplement: S1 Dataset — (ZIP) [file pone.0236452.s001.zip › Dataset/set-2/Adenoma/test8_231.jpg]

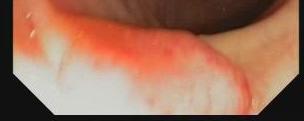

Supplement: S1 Dataset — (ZIP) [file pone.0236452.s001.zip › Dataset/set-2/Adenoma/test1_144.jpg]

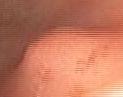

Supplement: S1 Dataset — (ZIP) [file pone.0236452.s001.zip › Dataset/set-2/Adenoma/test5_36.jpg]

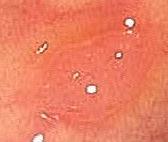

Supplement: S1 Dataset — (ZIP) [file pone.0236452.s001.zip › Dataset/set-2/Adenoma/test21_11.jpg]

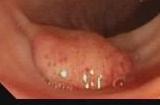

Supplement: S1 Dataset — (ZIP) [file pone.0236452.s001.zip › Dataset/set-2/Adenoma/test1_193.jpg]

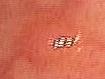

Supplement: S1 Dataset — (ZIP) [file pone.0236452.s001.zip › Dataset/set-2/Adenoma/test21_39.jpg]

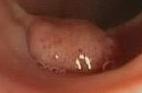

Supplement: S1 Dataset — (ZIP) [file pone.0236452.s001.zip › Dataset/set-2/Adenoma/test1_187.jpg]

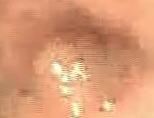

Supplement: S1 Dataset — (ZIP) [file pone.0236452.s001.zip › Dataset/set-2/Adenoma/test16_124.jpg]

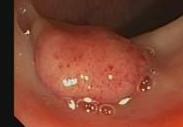

Supplement: S1 Dataset — (ZIP) [file pone.0236452.s001.zip › Dataset/set-2/Adenoma/test1_85.jpg]

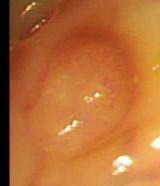

Supplement: S1 Dataset — (ZIP) [file pone.0236452.s001.zip › Dataset/set-2/Adenoma/test10_19.jpg]

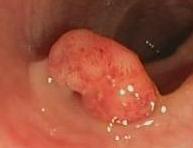

Supplement: S1 Dataset — (ZIP) [file pone.0236452.s001.zip › Dataset/set-2/Adenoma/test1_408.jpg]

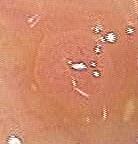

Supplement: S1 Dataset — (ZIP) [file pone.0236452.s001.zip › Dataset/set-2/Adenoma/test17_10.jpg]

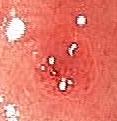

Supplement: S1 Dataset — (ZIP) [file pone.0236452.s001.zip › Dataset/set-2/Adenoma/test19_29.jpg]

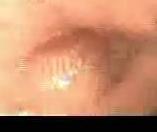

Supplement: S1 Dataset — (ZIP) [file pone.0236452.s001.zip › Dataset/set-2/Adenoma/test16_130.jpg]

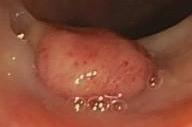

Supplement: S1 Dataset — (ZIP) [file pone.0236452.s001.zip › Dataset/set-2/Adenoma/test1_91.jpg]

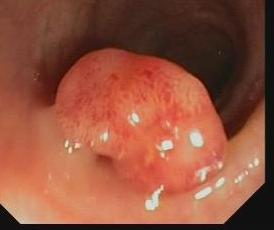

Supplement: S1 Dataset — (ZIP) [file pone.0236452.s001.zip › Dataset/set-2/Adenoma/test1_434.jpg]

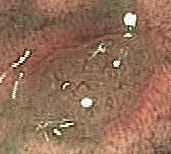

Supplement: S1 Dataset — (ZIP) [file pone.0236452.s001.zip › Dataset/set-2/Adenoma/test21_321.jpg]

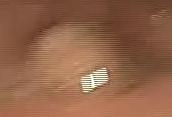

Supplement: S1 Dataset — (ZIP) [file pone.0236452.s001.zip › Dataset/set-2/Adenoma/test16_118.jpg]

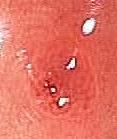

Supplement: S1 Dataset — (ZIP) [file pone.0236452.s001.zip › Dataset/set-2/Adenoma/test19_15.jpg]

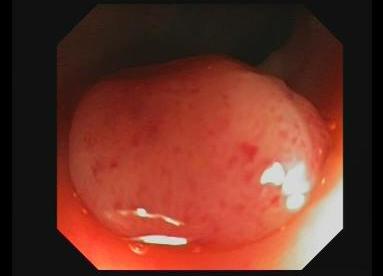

Supplement: S1 Dataset — (ZIP) [file pone.0236452.s001.zip › Dataset/set-2/Adenoma/test1_346.jpg]

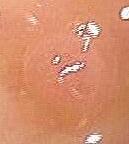

Supplement: S1 Dataset — (ZIP) [file pone.0236452.s001.zip › Dataset/set-2/Adenoma/test17_38.jpg]

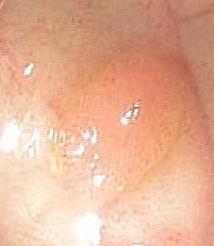

Supplement: S1 Dataset — (ZIP) [file pone.0236452.s001.zip › Dataset/set-2/Adenoma/test8_89.jpg]

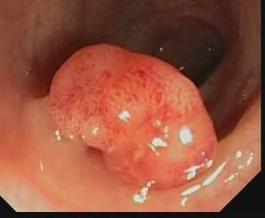

Supplement: S1 Dataset — (ZIP) [file pone.0236452.s001.zip › Dataset/set-2/Adenoma/test1_420.jpg]

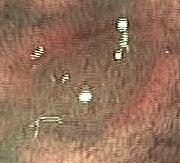

Supplement: S1 Dataset — (ZIP) [file pone.0236452.s001.zip › Dataset/set-2/Adenoma/test21_335.jpg]

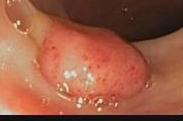

Supplement: S1 Dataset — (ZIP) [file pone.0236452.s001.zip › Dataset/set-2/Adenoma/test1_46.jpg]

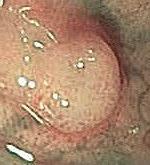

Supplement: S1 Dataset — (ZIP) [file pone.0236452.s001.zip › Dataset/set-2/Adenoma/test22_68.jpg]

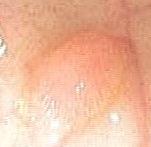

Supplement: S1 Dataset — (ZIP) [file pone.0236452.s001.zip › Dataset/set-2/Adenoma/test8_62.jpg]

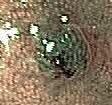

Supplement: S1 Dataset — (ZIP) [file pone.0236452.s001.zip › Dataset/set-2/Adenoma/test19_200.jpg]

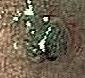

Supplement: S1 Dataset — (ZIP) [file pone.0236452.s001.zip › Dataset/set-2/Adenoma/test19_214.jpg]

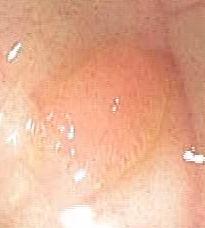

Supplement: S1 Dataset — (ZIP) [file pone.0236452.s001.zip › Dataset/set-2/Adenoma/test8_76.jpg]

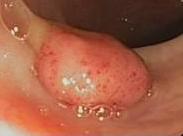

Supplement: S1 Dataset — (ZIP) [file pone.0236452.s001.zip › Dataset/set-2/Adenoma/test1_52.jpg]

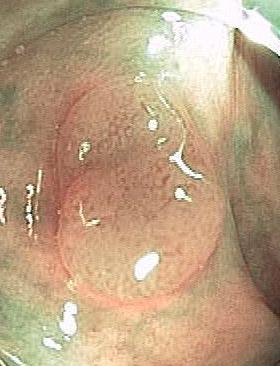

Supplement: S1 Dataset — (ZIP) [file pone.0236452.s001.zip › Dataset/set-2/Adenoma/test22_209.jpg]

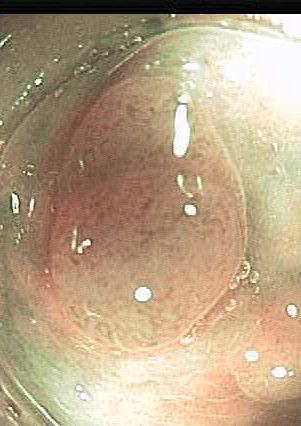

Supplement: S1 Dataset — (ZIP) [file pone.0236452.s001.zip › Dataset/set-2/Adenoma/test22_221.jpg]

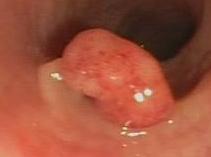

Supplement: S1 Dataset — (ZIP) [file pone.0236452.s001.zip › Dataset/set-2/Adenoma/test1_391.jpg]

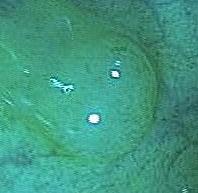

Supplement: S1 Dataset — (ZIP) [file pone.0236452.s001.zip › Dataset/set-2/Adenoma/test22_40.jpg]

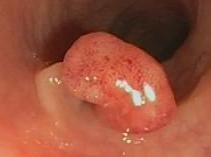

Supplement: S1 Dataset — (ZIP) [file pone.0236452.s001.zip › Dataset/set-2/Adenoma/test1_385.jpg]

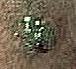

Supplement: S1 Dataset — (ZIP) [file pone.0236452.s001.zip › Dataset/set-2/Adenoma/test19_228.jpg]

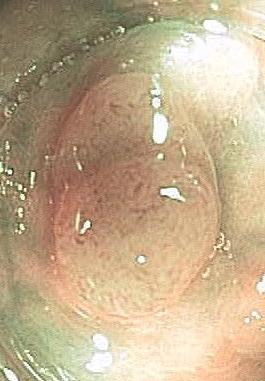

Supplement: S1 Dataset — (ZIP) [file pone.0236452.s001.zip › Dataset/set-2/Adenoma/test22_235.jpg]

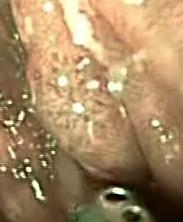

Supplement: S1 Dataset — (ZIP) [file pone.0236452.s001.zip › Dataset/set-2/Adenoma/test15_193.jpg]

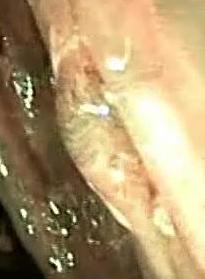

Supplement: S1 Dataset — (ZIP) [file pone.0236452.s001.zip › Dataset/set-2/Adenoma/test15_15.jpg]

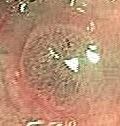

Supplement: S1 Dataset — (ZIP) [file pone.0236452.s001.zip › Dataset/set-2/Adenoma/test17_243.jpg]

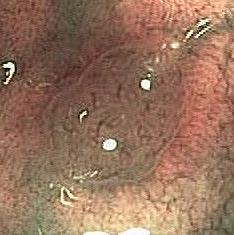

Supplement: S1 Dataset — (ZIP) [file pone.0236452.s001.zip › Dataset/set-2/Adenoma/test21_269.jpg]

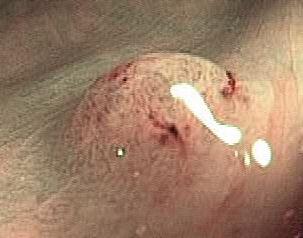

Supplement: S1 Dataset — (ZIP) [file pone.0236452.s001.zip › Dataset/set-2/Adenoma/test5_158.jpg]

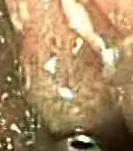

Supplement: S1 Dataset — (ZIP) [file pone.0236452.s001.zip › Dataset/set-2/Adenoma/test15_187.jpg]

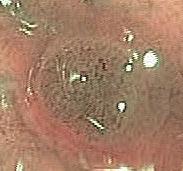

Supplement: S1 Dataset — (ZIP) [file pone.0236452.s001.zip › Dataset/set-2/Adenoma/test17_257.jpg]

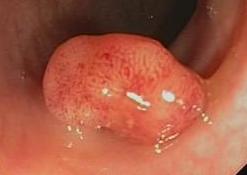

Supplement: S1 Dataset — (ZIP) [file pone.0236452.s001.zip › Dataset/set-2/Adenoma/test1_540.jpg]

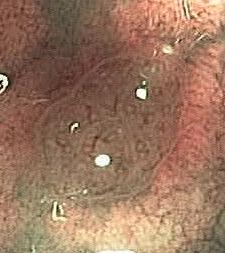

Supplement: S1 Dataset — (ZIP) [file pone.0236452.s001.zip › Dataset/set-2/Adenoma/test21_255.jpg]

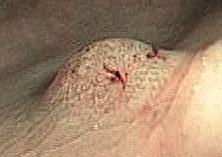

Supplement: S1 Dataset — (ZIP) [file pone.0236452.s001.zip › Dataset/set-2/Adenoma/test5_164.jpg]

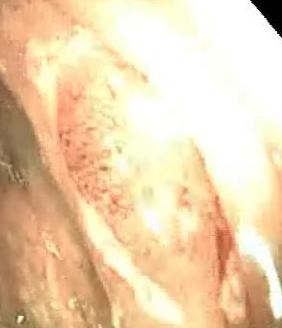

Supplement: S1 Dataset — (ZIP) [file pone.0236452.s001.zip › Dataset/set-2/Adenoma/test15_29.jpg]

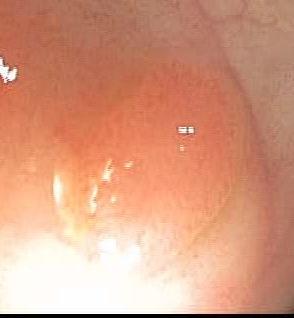

Supplement: S1 Dataset — (ZIP) [file pone.0236452.s001.zip › Dataset/set-2/Adenoma/test8_153.jpg]

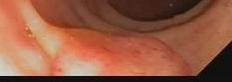

Supplement: S1 Dataset — (ZIP) [file pone.0236452.s001.zip › Dataset/set-2/Adenoma/test1_226.jpg]

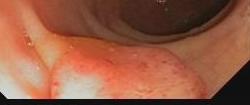

Supplement: S1 Dataset — (ZIP) [file pone.0236452.s001.zip › Dataset/set-2/Adenoma/test1_232.jpg]

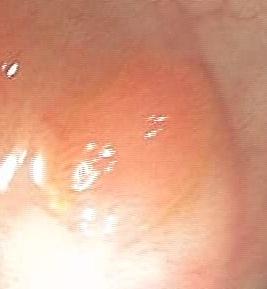

Supplement: S1 Dataset — (ZIP) [file pone.0236452.s001.zip › Dataset/set-2/Adenoma/test8_147.jpg]

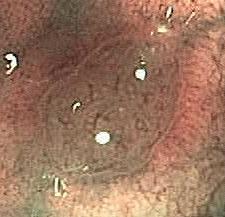

Supplement: S1 Dataset — (ZIP) [file pone.0236452.s001.zip › Dataset/set-2/Adenoma/test21_241.jpg]

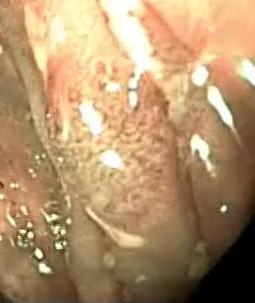

Supplement: S1 Dataset — (ZIP) [file pone.0236452.s001.zip › Dataset/set-2/Adenoma/test15_150.jpg]

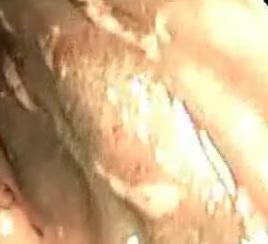

Supplement: S1 Dataset — (ZIP) [file pone.0236452.s001.zip › Dataset/set-2/Adenoma/test15_144.jpg]

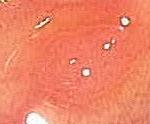

Supplement: S1 Dataset — (ZIP) [file pone.0236452.s001.zip › Dataset/set-2/Adenoma/test21_1.jpg]

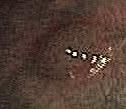

Supplement: S1 Dataset — (ZIP) [file pone.0236452.s001.zip › Dataset/set-2/Adenoma/test21_296.jpg]

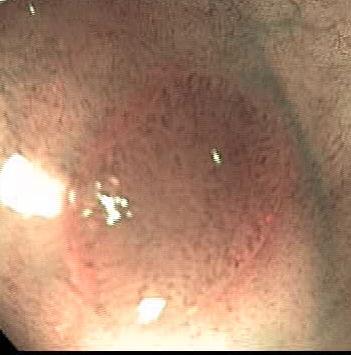

Supplement: S1 Dataset — (ZIP) [file pone.0236452.s001.zip › Dataset/set-2/Adenoma/test8_190.jpg]

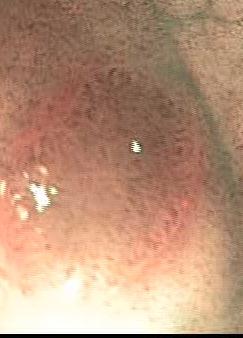

Supplement: S1 Dataset — (ZIP) [file pone.0236452.s001.zip › Dataset/set-2/Adenoma/test8_184.jpg]

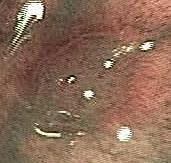

Supplement: S1 Dataset — (ZIP) [file pone.0236452.s001.zip › Dataset/set-2/Adenoma/test21_282.jpg]

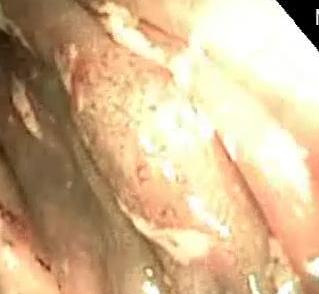

Supplement: S1 Dataset — (ZIP) [file pone.0236452.s001.zip › Dataset/set-2/Adenoma/test15_178.jpg]

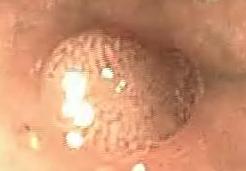

Supplement: S1 Dataset — (ZIP) [file pone.0236452.s001.zip › Dataset/set-2/Adenoma/test16_78.jpg]

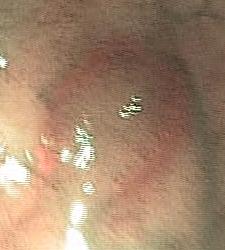

Supplement: S1 Dataset — (ZIP) [file pone.0236452.s001.zip › Dataset/set-2/Adenoma/test8_379.jpg]

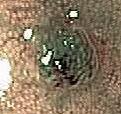

Supplement: S1 Dataset — (ZIP) [file pone.0236452.s001.zip › Dataset/set-2/Adenoma/test19_189.jpg]

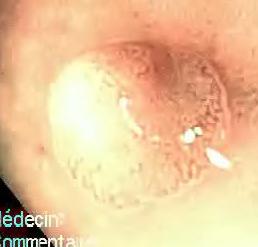

Supplement: S1 Dataset — (ZIP) [file pone.0236452.s001.zip › Dataset/set-2/Adenoma/test16_44.jpg]

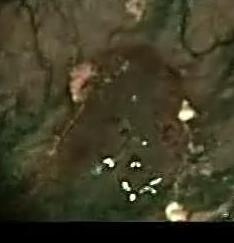

Supplement: S1 Dataset — (ZIP) [file pone.0236452.s001.zip › Dataset/set-2/Adenoma/test24_106.jpg]

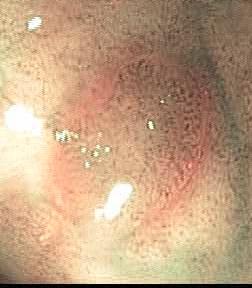

Supplement: S1 Dataset — (ZIP) [file pone.0236452.s001.zip › Dataset/set-2/Adenoma/test8_351.jpg]

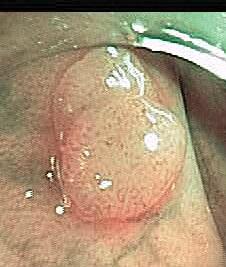

Supplement: S1 Dataset — (ZIP) [file pone.0236452.s001.zip › Dataset/set-2/Adenoma/test22_194.jpg]

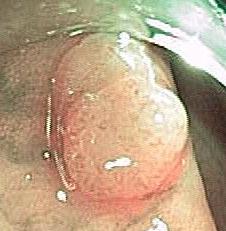

Supplement: S1 Dataset — (ZIP) [file pone.0236452.s001.zip › Dataset/set-2/Adenoma/test22_180.jpg]

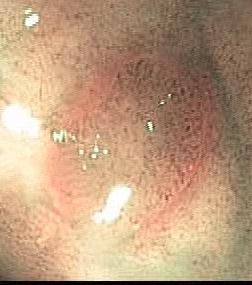

Supplement: S1 Dataset — (ZIP) [file pone.0236452.s001.zip › Dataset/set-2/Adenoma/test8_345.jpg]

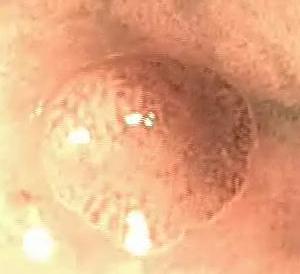

Supplement: S1 Dataset — (ZIP) [file pone.0236452.s001.zip › Dataset/set-2/Adenoma/test16_50.jpg]

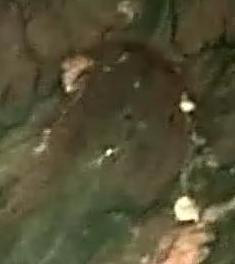

Supplement: S1 Dataset — (ZIP) [file pone.0236452.s001.zip › Dataset/set-2/Adenoma/test24_112.jpg]

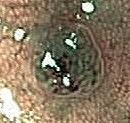

Supplement: S1 Dataset — (ZIP) [file pone.0236452.s001.zip › Dataset/set-2/Adenoma/test19_176.jpg]

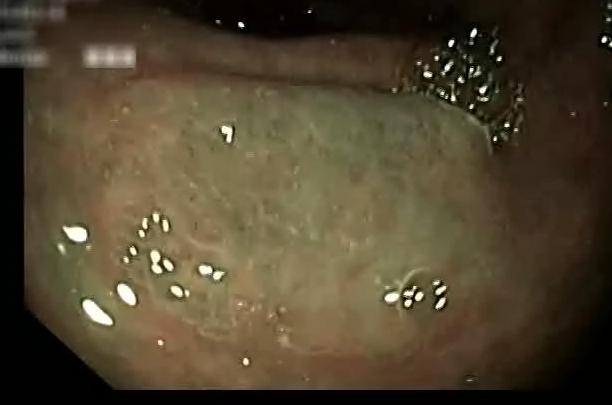

Supplement: S1 Dataset — (ZIP) [file pone.0236452.s001.zip › Dataset/set-2/Adenoma/test23_3.jpg]

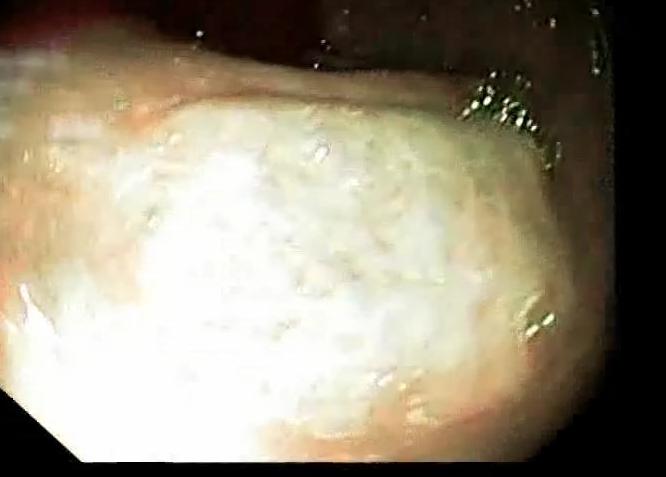

Supplement: S1 Dataset — (ZIP) [file pone.0236452.s001.zip › Dataset/set-2/Adenoma/test23_14.jpg]

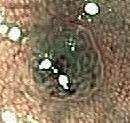

Supplement: S1 Dataset — (ZIP) [file pone.0236452.s001.zip › Dataset/set-2/Adenoma/test19_162.jpg]

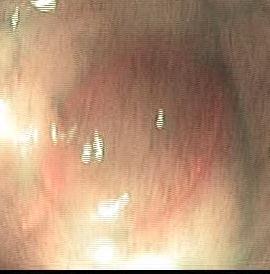

Supplement: S1 Dataset — (ZIP) [file pone.0236452.s001.zip › Dataset/set-2/Adenoma/test8_392.jpg]

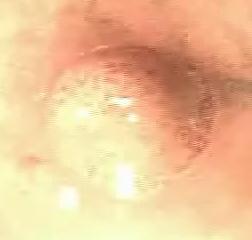

Supplement: S1 Dataset — (ZIP) [file pone.0236452.s001.zip › Dataset/set-2/Adenoma/test16_87.jpg]

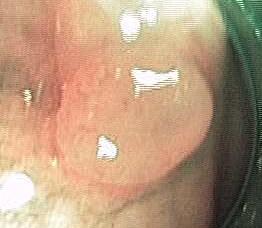

Supplement: S1 Dataset — (ZIP) [file pone.0236452.s001.zip › Dataset/set-2/Adenoma/test22_157.jpg]

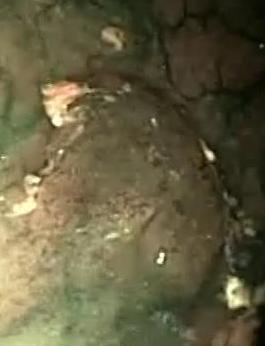

Supplement: S1 Dataset — (ZIP) [file pone.0236452.s001.zip › Dataset/set-2/Adenoma/test24_35.jpg]

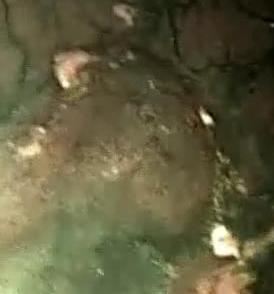

Supplement: S1 Dataset — (ZIP) [file pone.0236452.s001.zip › Dataset/set-2/Adenoma/test24_21.jpg]

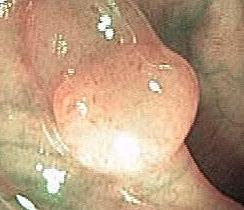

Supplement: S1 Dataset — (ZIP) [file pone.0236452.s001.zip › Dataset/set-2/Adenoma/test22_143.jpg]

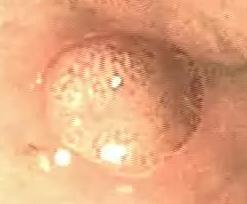

Supplement: S1 Dataset — (ZIP) [file pone.0236452.s001.zip › Dataset/set-2/Adenoma/test16_93.jpg]

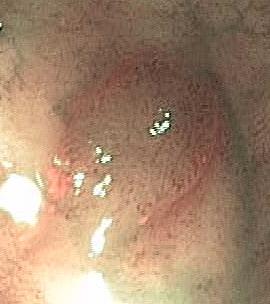

Supplement: S1 Dataset — (ZIP) [file pone.0236452.s001.zip › Dataset/set-2/Adenoma/test8_386.jpg]

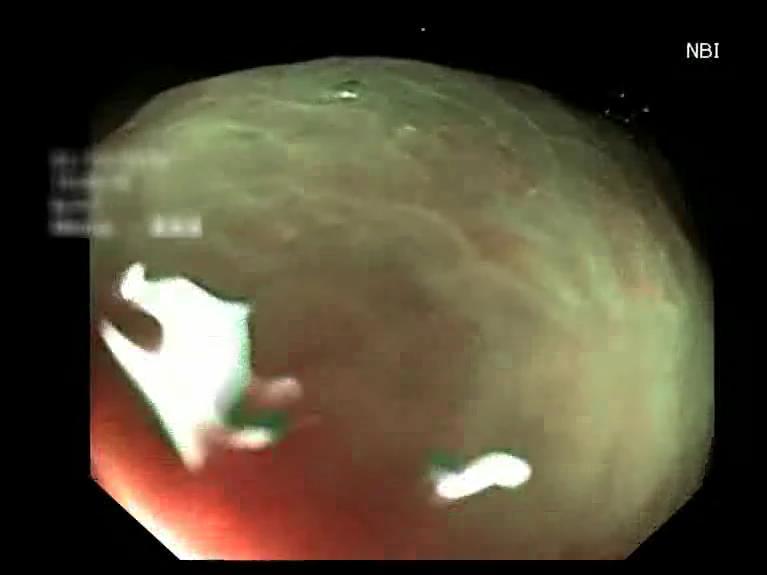

Supplement: S1 Dataset — (ZIP) [file pone.0236452.s001.zip › Dataset/set-2/Adenoma/test23_28.jpg]

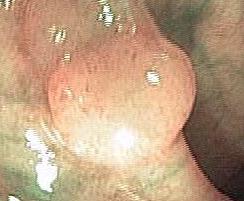

Supplement: S1 Dataset — (ZIP) [file pone.0236452.s001.zip › Dataset/set-2/Adenoma/test22_142.jpg]

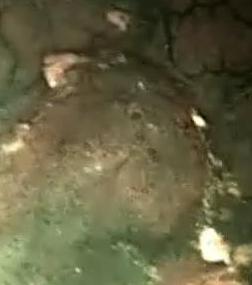

Supplement: S1 Dataset — (ZIP) [file pone.0236452.s001.zip › Dataset/set-2/Adenoma/test24_20.jpg]
